# Acute Acalculous Cholecystitis in the Setting of Negative Ultrasound and Computed Tomography Scan of the Abdomen

**DOI:** 10.7759/cureus.2243

**Published:** 2018-02-28

**Authors:** Muhammad Shafiq, Yousaf Zafar

**Affiliations:** 1 Department of Internal Medicine, University of Missouri Kansas City (UMKC)

**Keywords:** acute acalculous cholecystitis, computed tomography scanning, hepatobiliary iminodiacetic acid scan, ultrasound abdomen

## Abstract

Acute acalculous cholecystitis (AAC) is most commonly seen after surgery in critically ill patients. Early diagnosis and treatment is the key in the management of AAC. Ultrasound is the commonly used first modality for right upper quadrant (RUQ) pain with sensitivity equal to or greater than 80% for AAC. Computed tomography (CT) scan is reported to have a sensitivity close to 90% and if both the ultrasound and CT scan are combined, it further increases the sensitivity for the diagnosis of AAC. It is unlikely for AAC to be present in the setting of both negative ultrasound and CT scan of the abdomen. Our case report presents a similar clinical scenario where the patient was found to have both negative ultrasound and CT scan abdomen but was positive on hepatobiliary iminodiacetic acid (HIDA) scan for AAC as stated below.

A 32-year-old male presented to the emergency room with complaints of RUQ pain for two days which was associated with one episode of non-bilious and non-bloody vomiting as well as subjective fever and chills. On presentation, the patient's blood pressure was 87/54 mmHg. Other vitals were unremarkable.

The patient had both CT scan abdomen and ultrasound of the RUQ done which reported non-specific findings but were grossly negative for AAC. On the first night of admission, the patient's blood pressure dropped to 84/32 mmHg. The patient was transferred to the intensive care unit (ICU) given the concern for sepsis and was started on intravenous (IV) vancomycin, IV metronidazole and IV levofloxacin (patient was allergic to penicillin). Given the high clinical suspicion, a HIDA scan performed which was positive for AAC. The patient then had a cholecystostomy tube placed by the interventional radiology team. The patient improved rapidly and was eventually discharged with a 14-day course of Bactrim DS (Roche Pharmaceuticals, Nutley, NJ) and metronidazole, and four weeks of outpatient follow up with general surgery. The patient underwent outpatient cholecystectomy in the eighth week from discharge.

This leads to the conclusion that even if both the ultrasound and CT scan of the abdomen are negative and clinical suspicion is still high for AAC, the patient should undergo a HIDA scan as delay in treatment is associated with greater than 50% mortality in patients with AAC.

## Introduction

Acute acalculous cholecystitis (AAC) is an acute inflammatory condition of the gall bladder in the absence of gall stones. It is the result of multifactorial pathogenesis and is most commonly seen after surgery and in critically ill patients [1-2]. Early diagnosis and treatment is the key in the management of AAC because a delay in treatment is associated with higher morbidity and mortality as evident in one review study which reports an overall mortality of 33.3% [3].

There is conflicting data about the significance of ultrasound in the diagnosis of AAC. Some studies report the sensitivity of the ultrasound to be equal to or more than 80% in the diagnosis of AAC [1, 4] but other studies report the ultrasound to have either a sensitivity less than 70% [5] or showing non-specific findings of unknown clinical significance [6]. Still, ultrasound is one of the first diagnostic modalities used in the diagnosis of AAC given its easy availability and cost-effectiveness. In order to evaluate for complications or to rule out alternative etiologies, a computed tomography (CT) scan of the abdomen is often obtained [7]. CT scan of the abdomen has been reported to have a sensitivity equal to or greater than 90% in the diagnosis of AAC [1, 4].

Combining the ultrasound and CT scan abdomen significantly increases the sensitivity for the diagnosis of AAC [7] yet, the diagnosis of AAC can be missed even if both modalities are used together. This has been documented in one case report [8]. We report a similar scenario where both the ultrasound and CT scan of the abdomen (performed during the same hospitalization period for a similar clinical presentation of sepsis and right upper quadrant (RUQ) pain) failed to diagnose AAC.

## Case presentation

Materials and Methods

The authors performed a search on PubMed using Medical Subject Heading (MeSH) terms of acalculous cholecystitis, CT scan, ultrasound abdomen, and hepatobiliary iminodiacetic acid (HIDA) scan. This case report has also been presented as a poster at the American College of Gastroenterology, Annual Scientific Meeting in October 2017, at Orlando, Florida, USA.

Clinical Presentation

We report the case of a 32-year-old male who presented to the emergency room of the hospital for the chief complaint of RUQ pain for two days. This abdominal pain was associated with one episode of non-bilious and non-bloody vomiting. The patient also reported subjective fever and chills. Last bowel movement was one day prior to presentation.

The patient denied any diarrhea, constipation, any sick contacts, recent travel, or any alcohol abuse. However, the patient had past medical history of steatohepatitis, gastroparesis status post (s/p) gastrostomy tube, and un-controlled seizures s/p multiple surgical procedures including vagal stimulator and multiple brain resections and was on five anti-seizure medications (including Keppra® 2 gram orally twice a day, Depakote® 250 milligram orally every eight hours, carbamazepine 500 milligram orally three times a day, Onfi® 15 milligram orally two times a day and Vimpat® 200 milligram orally two times a day).

According to the family, the patient had a fall following a seizure two days prior to the presentation. The patient's family attributed his abdominal pain to the fall. 

On presentation, the patient's vitals were temperature 98°F, respiratory rate 18/minute, blood pressure 87/54 mmHg, pulse 95/minute, and pulse oximetry 98% on room air.

In the emergency room, the patient had laboratory blood tests done which showed a normal white blood cells (WBC) count of 9.32 TH/uL but alkaline phosphatase was 70 IU/L, aspartate aminotransferase was 542 IU/L, alanine transaminase was 217 IU/L, serum albumin was 4.1 g/dL, and total bilirubin was 1.9 mg/dL. Lipase was within the normal range. The lactate level was slightly elevated at 3.1 mmol/L. The laboratory reference range for lactate is 0.0-2.0 mmol/L. Hepatitis A, B, and C serologies were negative.

**Figure 1:**
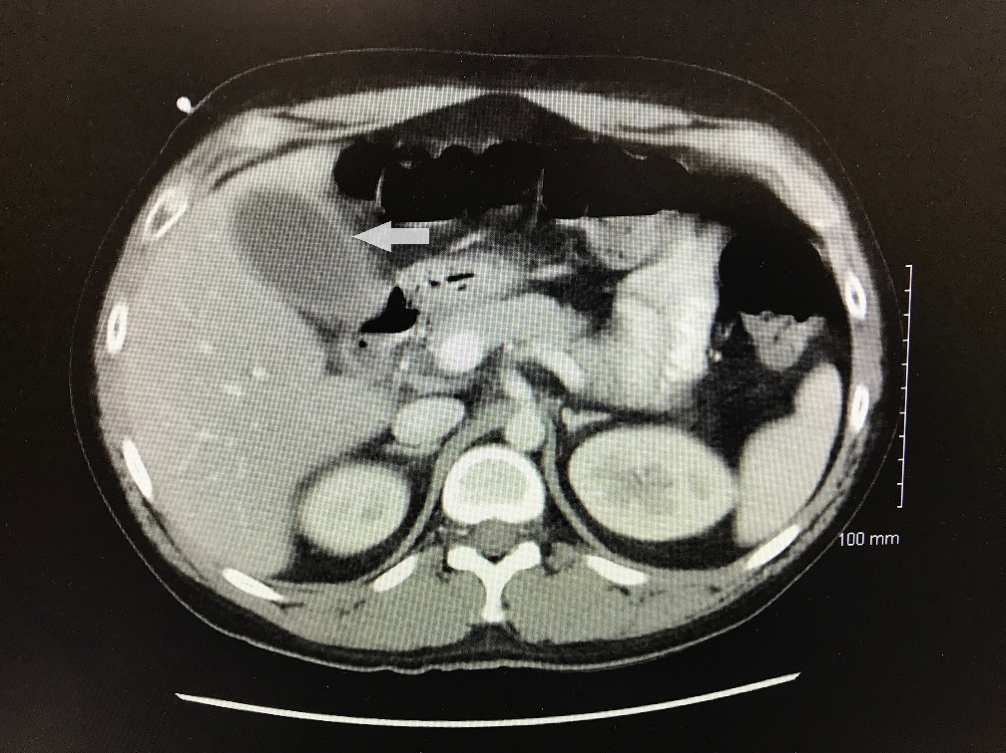
Computed tomography scan of abdomen Arrow indicating non-specific findings of peri-cholecystic fluid without any signs of gall stones.

The patient also underwent CT scan of the abdomen in the emergency room Figure 1).

The results of the CT scan were as follows: 1) Distended gallbladder with surrounding pericholecystic fluid likely relates to underlying hepatitis; RUQ ultrasound can be obtained for further evaluation. 2) Segmental pneumatosis in the ascending colon is likely benign in etiology given normal enhancement and lack of bowel wall thickening. 3) Enteric tube terminates in the jejunum with contrast in the jejunal loops; no contrast extravasation. 4) Stable enhancing lesions in the left lower lobe of the liver favor focal nodular hyperplasia over adenoma.

Based on these results, the patient then also had an ultrasound of the RUQ. The following observations were made: 1) Sludge within the gall bladder; no evidence of cholecystitis. 2) Hepatic steatosis. 3) Stable hypoechoic lesions within the liver which favor focal nodular hyperplasia over adenoma.

The patient was initially admitted to the medicine floor. On hospital day one at midnight, the patient became hypotensive with a blood pressure of 84/32 mmHg and WBC count trended up to 12 TH/uL. Given the concern for sepsis, the patient was transferred to intensive care unit (ICU). The patient was started on intravenous (IV) vancomycin, IV metronidazole and IV levofloxacin (patient was allergic to penicillin). Repeat blood cultures were drawn from two different sites; 30 minutes apart from each other. Arterial line and central line were placed but the patient did not require any pressor and responded well to initial fluid resuscitation.

**Figure 2:**
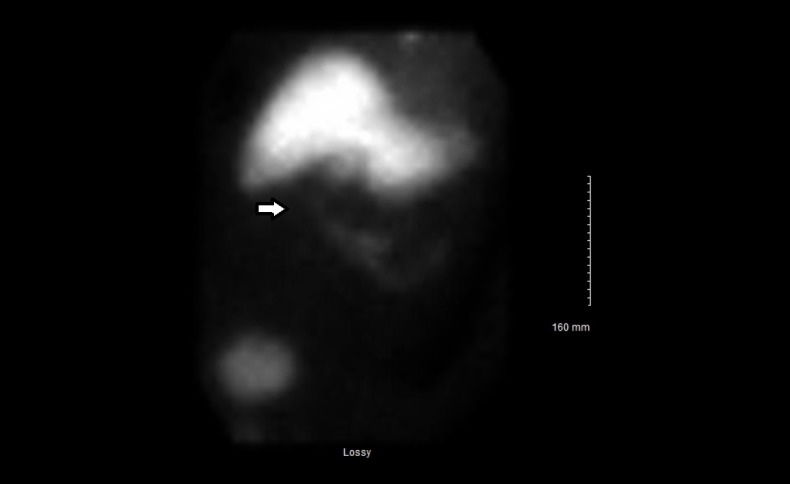
Hepatobiliary iminodiacetic acid (HIDA) scan The arrow indicates the gall bladder area which doesn't light up with the radio-tracer.

General surgery was consulted and on the second day, the patient had a positive Murphy's sign on physical exam. Given the high clinical suspicion for acute cholecystitis despite the negative ultrasound and CT scan, general surgery ordered a HIDA scan. The HIDA scan was completed on hospital day three (Figure 2).

The result of the HIDA Scan was non-visualization of the gall bladder concerning for cystic duct obstruction/acute cholecystitis. Surgical consultation was advised.

Given the HIDA scan results, interventional radiology was consulted on the fourth hospital day and they placed the cholecystostomy tube. The patient improved significantly and was transferred out of the ICU. The plan per the general surgery team was to keep the cholecystostomy tube for four weeks and then follow up as an outpatient. All cultures (including blood cultures) remained negative; therefore, the patient was eventually discharged on oral antibiotics (Bactrim DS® and metronidazole) for a total of 14 days. The patient finally had outpatient cholecystectomy done eight weeks from discharge and the cholecystostomy tube was removed.

## Discussion

Ultrasound is usually the first modality used, given its easy accessibility and cost-effectiveness, for the diagnosis of AAC regardless of the type; however, there are known reports of false negatives with ultrasound in a patient suffering from AAC [5-6]. A CT scan of the abdomen is not routinely used for the diagnosis of AAC; it is used when there is a suspicion for alternative diagnosis or complications. Yet, a CT scan is reported to have sensitivity equal to or greater than 90% [1, 4] in the diagnosis of AAC. If the CT scan and ultrasound are combined together, it further enhances the sensitivity in the diagnosis of AAC [7]. In our case presentation, it is evident that both the CT scan and ultrasound were performed but still, the diagnosis of AAC was delayed as it was missed by both imaging systems which reported only non-specific findings.

This leads to the conclusion that if the clinical suspicion for AAC is high, the patient should undergo HIDA scan even if both the ultrasound and CT scan are negative. HIDA scan is reported to have a specificity of more than 95% [9-10] and this test has been validated for decades now.

## Conclusions

Early diagnosis and treatment is the key to the management of AAC because if the treatment is delayed, the patient mortality increases to more than 50%. Therefore, even if both the ultrasound and CT scan of the abdomen are negative and clinical suspicion for AAC is high, the patient should undergo a HIDA scan.

## References

[1] Barie PS, Eachempati SR (2010). Acute acalculous cholecystitis. Gastroenterol Clin North Am.

[2] Cornwell EE, Rodriguez A, Mirvis SE, Shorr RM (1989). Acute acalculous cholecystitis in critically injured patients. Preoperative diagnostic imaging. Ann Surg.

[3] DuPriest RW, Khaneja SC, Cowley RA (1979). Acute cholecystitis complicating trauma. Annals of Surgery.

[4] Mirvis SE, Vainright JR, Nelson AW (1986). The diagnosis of acute acalculous cholecystitis: a comparison of sonography, scintigraphy, and CT. AJR Am J Roentgenol.

[5] Molenat F, Boussuges A, Valantin V, Sainty JM (1996). Gallbladder abnormalities in medical ICU patients: an ultrasonographic study. Intensive Care Med.

[6] Boland GW, Slater G, Lu DS, Eisenberg P, Lee MJ, Mueller PR (2000). Prevalence and significance of gallbladder abnormalities seen on sonography in intensive care unit patients. AJR Am J Roentgenol.

[7] Blankenberg F, Wirth R, Jeffrey RB Jr, Mindelzun R, Francis I (1991). Computed tomography as an adjunct to ultrasound in the diagnosis of acute acalculous cholecystitis. Gastrointest Radiol.

[8] Fenakel G, Schattner A (1994). Acute acalculous cholecystitis (Article in Hebrew). Harefuah.

[9] Mariat G, Mahul P, Prevot N (2000). Contribution of ultrasonography and cholescintigraphy to the diagnosis of acute acalculous cholecystitis in intensive care unit patients. Intensive Care Med.

[10] Prevot N, Mariat G, Mahul P (1999). Contribution of cholescintigraphy to the early diagnosis of acute acalculous cholecystitis in intensive-care-unit patients. Eur J Nucl Med.

